# Complete Genome of Sphingomonas aerolata PDD-32b-11, Isolated from Cloud Water at the Summit of Puy de Dôme, France

**DOI:** 10.1128/mra.00684-22

**Published:** 2022-09-15

**Authors:** Domitille Jarrige, Thierry Nadalig, Muriel Joly, Martine Sancelme, Stéphane Vuilleumier, Pierre Amato, Françoise Bringel

**Affiliations:** a Génétique Moléculaire, Génomique, Microbiologie (GMGM), Université de Strasbourg, Strasbourg, France; b Université Clermont Auvergne, Clermont Auvergne Institut National Polytechnique (INP), Centre National de la Recherche Scientifique (CNRS), Institut de Chimie de Clermont-Ferrand (ICCF), Clermont-Ferrand, France; University of Southern California

## Abstract

The complete genome of Sphingomonas aerolata PDD-32b-11, a bacterium isolated from cloud water, was sequenced. It features four circular replicons, a chromosome of 3.99 Mbp, and three plasmids. Two putative rhodopsin-encoding genes were detected which might act as proton pumps to harvest light energy.

## ANNOUNCEMENT

Metabolically active microorganisms in clouds participate in atmospheric chemistry (1, 2). Genome analysis of atmospheric bacteria may help identify key functions involved in the maintenance of activity under harsh conditions (e.g., low nutrient and water levels, rapid variations in temperature, and UV exposure), and their impact on atmospheric functioning. Members of the *Sphingomonas* genus (3) have been isolated from air in both natural and anthropized environments (4–7). Representatives were regularly isolated from clouds sampled at the summit of puy de Dôme (1465 m above sea level) (8). Among those, strain PDD-32b-11 was isolated in 2009 (8).

**FIG 1:**
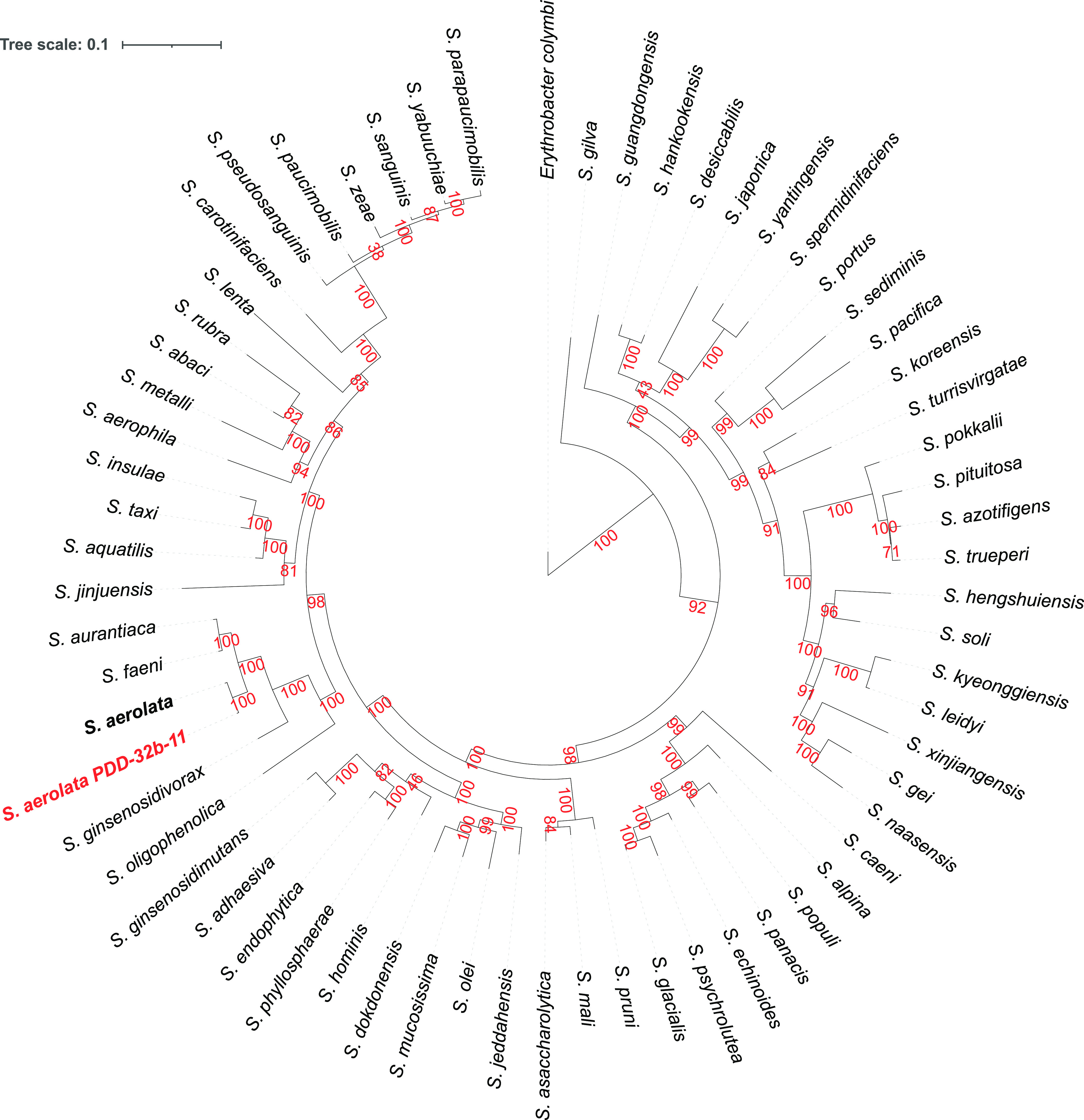
Phylogenetic tree of the *Sphingomonas* genus. The approximately maximum likelihood tree was built using GTDB-Tk (11) with 118 marker genes, pruned with ETE v3 (16), and visualized with ITOL v 6.5.8 (17). Erythrobacter colymbi was chosen as the outgroup. Bootstrap support values in percent are indicated in red.

The complete genome of strain PDD-32b-11 was sequenced by single-molecule real-time long reads using a PacBio Sequel II sequencer (Pacific Biosciences, Menlo Park, CA, USA; Gentyane sequencing platform, Clermont-Ferrand, France). Genomic DNA was extracted from an aerobic culture grown at 17°C in R2A medium (Millipore) using the MasterPureTM complete DNA & RNA purification kit (Lucigen). A SMRTbell express 2 template prep kit was used for library preparation from sheared DNA fragments of approximately 10 kb on average. A SMRTBell polymerase complex was obtained using Binding kit 2.2 (Pacific Biosciences) and primer v5 was sequenced using a PacBio SMRTcell 8 M and a sequencing plate 2.0. Default parameters were used with all software unless otherwise specified. Raw sequencing data (CCS reads) were treated using the library smrttools10.1.0.119588 (Pacific Biosciences) (3 minimum passes for “ccs” tool; split-bam-named, ccs and peek-guess for “lima” tool). The resulting 70391 reads (mean read length 7119 nt) were assembled with Flye v2.9 (9) (parameters: pacbio-hifi, genome-size 5 Mbp), yielding a genome of 4,209,937 bp in total, with four circular contigs (Table 1). The chromosome (3.99 Mbp) harbors all rRNA and tRNA encoding genes. The three other contigs (39, 77, and 102 kb) have a lower GC content (59 to 62%) compared to the chromosome (66%) and encode replication initiator proteins typical of plasmid replication. Genome assembly completeness was assessed with BUSCO v5.2.2 (10) (reference data set sphingomonadales_odb10 2021-02-23; 124 genomes; 1018 BUSCOs). Strain PDD-32b-11 was assigned to the species Sphingomonas aerolata using GTDB-Tk v2.0.0 (GTDB r207) (11) (Fig. 1).

**TABLE 1:** Sphingomonas aerolata PDD-32b-11 genome assembly and annotation statistics

Feature	Sphingomonas aerolata PDD-32b-11
Sequencing coverage depth	119×
Assembly size (Mbp)	4.21
No. of scaffolds/contigs	4
GC content (%)	66.09
Chromosome contig (bp)	3,991,634
Plasmids contigs (bp)	38,945; 77,112; 102,246
Annotation statistics	
No. of predicted CDS^*a*^	4,181
No. of predicted tRNAs	57 (chromosome-encoded)
No. of predicted rRNAs	12 (chromosome-encoded)
Average CDS length (bp)	919.67
BUSCO v5.2.2 assembly completeness assessment (%)	
Complete	99.5
Single	99.3
Duplicated	0.2
Fragmented	0.2
Missing	0.3

a CDS, Coding sequence.

The obtained genome assembly was annotated using the NCBI Prokaryotic Genome Annotation Pipeline v6.1 (12). Additional putative genes were annotated on the MicroScope platform v3.15.4 (13). Genes were identified for both tetrahydrofolate- (FolD and Fhs) and glutathione-dependent (FrmA and FrmC) pathways for oxidation of formaldehyde, a key compound of atmospheric chemistry (2). A partial photosynthesis gene cluster (PGC) (*bchI*, *bchD*, *bchO*, *bchF*, *bchG*, *ppaA*, *ppsR,* and *tspO*) typical of aerobic anoxygenic phototrophs (14) was also found. In addition, two putative homologs of the functional proton-pumping DTG/DTS rhodopsin of Pseudomonas putida (15) were identified, each adjacent to a gene encoding beta-carotene 15,15′-dioxygenases associated with biosynthesis of the retinal cofactor of light-driven rhodopsin proton pumps.

### Data availability.

The Sphingomonas aerolata PDD-32b-11 complete genome project was deposited in DDBJ/ENA/GenBank under accession numbers CP098762 to CP098765 within BioProject PRJNA847404. Raw reads were deposited in the Sequence Read Archive under accession number SRR19594883. Genome sequence and annotation are accessible on the MicroScope platform (https://mage.genoscope.cns.fr/microscope/genomic/overview.php?O_id=17210).
